# Embryo production by intracytoplasmic injection of sperm retrieved from Meishan neonatal testicular tissue cryopreserved and grafted into nude mice

**DOI:** 10.1111/asj.13138

**Published:** 2018-12-06

**Authors:** Hiroyuki Kaneko, Kazuhiro Kikuchi, Nguyen Thi Men, Junko Noguchi

**Affiliations:** ^1^ Institute of Agrobiological Sciences National Agriculture and Food Research Organization (NARO) Tsukuba Ibaraki Japan; ^2^ The United Graduate School of Veterinary Science Yamaguchi University Yamaguchi Japan

**Keywords:** blastocyst formation, cryopreservation, Meishan testis, sperm production, testicular xenografting

## Abstract

Testicular xenografting, combined with cryopreservation can assist conservation of the genetic diversity of indigenous pigs by salvaging germ cells from their neonatal testes. Using Meishan male piglets as an example, we examined whether testicular tissue would acquire the ability to produce sperm after cryopreservation and grafting into nude mice (MS group). For comparison, testicular tissue from neonatal Western crossbreed male piglets was used (WC group). Sixty days after xenografting (day 0 = grafting), MS grafts had already developed seminiferous tubules containing sperm, whereas in the WC grafts, sperm first appeared on day 120. The proportion of tubules containing spermatids and sperm was higher in the MS group than in the WC group between days 90 and 120. Moreover, in vitro‐matured porcine oocytes injected with a single sperm obtained from the MS group on day 180 developed to the blastocyst stage. The blastocyst formation rate after injection of the xenogeneic sperm was 14.6%, whereas the ratio in the absence of such injection (attributable to parthenogenesis) was 6.7%. Thus, cryopreserved Meishan testicular tissue acquired spermatogenic activity in host mice 60 days earlier than Western crossbreed tissue. Such xenogeneic sperm are likely capable of generating blastocysts in vitro.

## 1 INTRODUCTION

Grafting of testicular tissue into immunodeficient mice is a promising method of harvesting sperm from different donor species that have not yet reached sexual maturation (Arregui & Dobrinski, 2014; Kikuchi et al., 2016). Even after cryopreservation, fetal or neonatal testicular tissues from several donor species acquire full spermatogenic activity in host mice, their germ cells becoming elongated spermatids (neonatal pig: Caires, Schmidt, Oliver, de Avila, & McLean, 2008; sheep: Pukazhenthi et al., 2015) or sperm (neonatal rabbit: Shinohara et al., 2002; pig: Abrishami, Anzar, Yang, & Honaramooz, 2010; Honaramooz et al., 2002; Kaneko et al., 2013; fetal pig: Kaneko, Kikuchi, Men, et al., 2017; Kaneko, Kikuchi, Nakai, et al., 2017). The ability of xenogeneic sperm to generate offspring has been demonstrated by intracytoplasmic sperm injection (ICSI) using sperm from cryopreserved testicular tissues of neonatal rabbits (Shinohara et al., 2002) and fetal (Kaneko, Kikuchi, Nakai, et al., 2017) and neonatal pigs (Kaneko et al., 2013). Furthermore, the reproductive ability of offspring produced using such xenogeneic sperm has also been confirmed in pigs (Kaneko et al., 2014). Thus, testicular xenografting, combined with cryopreservation and ICSI is an effective method of storing and utilizing germ cells from immature animals, unlike conventional reproductive methods using fully differentiated germ cells obtained from sexually mature animals. These techniques are therefore probably applicable to rescue genetic information from valuable immature pigs that die quite young. Alternatively, systematic collection of neonatal testes might be an aid to conserve indigenous pigs whose populations are often small, but which possess unique phenotypes, thus having the potential to maintain the genetic diversity of pig species. As an example of the former application, we have generated progeny using fetal testis obtained from cloned pigs harboring a disruption of the X chromosome‐linked coagulation factor VIII (F8) gene (hemophilia‐A pig) (Kaneko, Kikuchi, Nakai, et al., 2017): such female cloned pigs (*F8* ^*+/−*^), despite having a recessive X‐linked condition, died of severe bleeding at an early age (Kaneko, Kikuchi, Nakai, et al., 2017), as was the case for male cloned pigs (*F8* ^*−/Y*^) (Kashiwakura et al., 2012). However, the above studies used testicular tissue from breeds of Western origin (Abrishami et al., 2010; Caires et al., 2008; Honaramooz et al., 2002; Kaneko et al., 2013; Kaneko, Kikuchi, Men, et al., 2017; Kaneko, Kikuchi, Nakai, et al., 2017).

The Meishan pig is a Chinese indigenous breed noted for early sexual maturity (Lunstra, Ford, Klindt, & Wise, 1997) and high prolificacy (Haley & Lee, 1993). In Meishan boars, sperm appear in the lumina of seminiferous tubules by 60 days of age (Harayama, Nanjo, Kanda, & Kato, 1991; Lunstra et al., 1997), 60–90 days earlier than in Western breeds (4–5 months of age, FlorCruz & Lapwood, 1978; van Straaten & Wensing, 1977). On the other hand, testis weight at onset of sperm production is lower in Meishan boars (40–60 g/paired testis weight, Harayama et al., 1991; Lunstra et al., 1997) than in Western breeds (100–250 g/paired testis weight, FlorCruz & Lapwood, 1978; van Straaten & Wensing, 1977). Thus, testis growth and differentiation in indigenous pigs are not always similar to those in Western breeds, and few studies of testis transplantation using indigenous boars have been reported.

In the present study, as an example of an indigenous pig breed, we examined whether testicular tissue from Meishan piglets would exhibit complete spermatogenic activity after cryopreservation and grafting into nude mice. We then injected sperm recovered from the host mice into in vitro‐matured porcine oocytes and assessed the competence of these oocytes to develop to the blastocyst stage.

## 2 MATERIALS AND METHODS

### 2.1 Experimental animals

All experiments were performed in accordance with protocols approved by the Animal Care Committee (# H18‐008‐02) of the Institute of Agrobiological Sciences, National Agriculture and Food Research Organization (NARO), Tsukuba, Japan. Meishan (MS) and Western crossbreed (WC, Landrace × Large White × Duroc) pigs used in this study were produced and reared according to the Japanese Feeding Standard for Swine at the Institute of Livestock and Grassland Science of NARO. Male nude mice (Crlj:CD1‐*Foxn1* ^*nu*^) 5–6 weeks old purchased from Charles River Japan (Yokohama, Japan) were kept in an environmentally controlled room maintained at a temperature at 24°C and 70% humidity, and illuminated daily from 05:00 to 19:00.

### 2.2 Chemicals

All chemicals were purchased from the Sigma‐Aldrich Corporation (St. Louis, MO, USA), unless otherwise indicated.

### 2.3 Vitrification of testicular tissue

Twelve MS piglets aged 5–16 days and 11 WC piglets aged 9–12 days were used as testis donors. After removing the capsule of each testis, the testicular parenchyma was minced into fragments measuring approximately 1.5 × 1.5 × 1.5 mm in saline supplemented with 660 units/ml penicillin G potassium and 0.2 mg/ml streptomycin sulfate at 25°C (Kaneko, Kikuchi, Nakai, & Noguchi, 2008; Kaneko et al., 2013; Kaneko, Kikuchi, Men, et al., 2017; Kaneko, Kikuchi, Nakai, et al., 2017). Immediately after mincing, the testicular fragments were vitrified according to the method described by Dinnyés, Dai, Jiang, and Yang (2000) and Somfai et al. (2007), with some modifications (Kaneko et al., 2013; Kaneko, Kikuchi, Men, et al., 2017; Kaneko, Kikuchi, Nakai, et al., 2017). Briefly, approximately 35 fragments were washed as a group, two times in 2 ml of base solution at room temperature (BS: IVC‐PyrLac solution [Kikuchi et al., 2002] supplemented with 20 mmol/L HEPES [Dojindo, Kumamoto, Japan]), and then transferred to 500 μl of equilibration solution (BS supplemented with 4% ethylene glycol [EG]) and incubated for 15 min. The equilibrated fragments were immersed in 500 μl of vitrification solution (BS supplemented with 35% EG, 5% polyvinyl pyrrolidone (Mr 40,000) and 0.3 mol/L trehalose) for 10 min. Each fragment was aspirated into a glass capillary, and dropped onto an aluminum foil boat partially immersed in liquid nitrogen with a minimum volume of vitrification solution to form a micro‐droplet. Finally, the vitrified droplets were placed in 2‐ml cryotubes (Corning 430488, Thermo Fisher Scientific, Waltham, MA, USA) immersed in liquid nitrogen using cooled forceps, and then the cryotubes were plunged into liquid nitrogen. The vitrified tissues were stored in liquid nitrogen until used for grafting.

### 2.4 Xenografting of testicular tissue

MS‐vitrified droplets after storage for between 2 and 20 months and WC‐vitrified droplets stored for 5.4 years were transferred to a warming solution (BS supplemented with 0.4 mol/L trehalose) at 37°C for 2 min (Kaneko et al., 2013; Kaneko, Kikuchi, Men, et al., 2017; Kaneko, Kikuchi, Nakai, et al., 2017). The testicular fragments were consecutively transferred for 2‐min periods to BS supplemented with 0.2, 0.1 and 0.05 mol/L trehalose to remove the cryoprotectants, and finally incubated in saline supplemented with antibiotics for at least 2 min at room temperature. Five fragments were randomly selected from those obtained from 5 individual MS donors and 5–8 fragments from 4 individual WC donors, and then fixed in Bouin's solution: these were used to obtain histological data for day 0 (day 0 = grafting).

Fifty‐four male nude mice aged 5–6 weeks were assigned to receive testicular tissues obtained from MS (MS group, *n* = 28) and WC piglets (WC group, *n* = 26). All mice were anesthetized with isoflurane (Intervet, Tokyo, Japan), and then castrated. Immediately after castration, 23–25 testicular fragments were inserted under the skin of each mouse within 30 min after the end of warming (Kaneko et al., 2013; Kaneko, Kikuchi, Men, et al., 2017; Kaneko, Kikuchi, Nakai, et al., 2017).

### 2.5 Recovery of grafts and sperm

Host mice in both groups were euthanatized by cervical dislocation under anesthesia with isoflurane on days 60, 90, 120 and 180. All visible testicular grafts were immediately recovered from the host mice and placed in collection medium (Dulbecco's PBS; Nissui, Tokyo, Japan, supplemented with 5 mg/mL BSA) at 37°C and then weighed. Three pieces were excised from the different larger grafts in each mouse and fixed in Bouin's solution for histological examination. The remaining portions were cut into small pieces in the collection medium, and the presence of sperm released into the medium was recorded.

For ICSI, sperm were collected from two hosts in the MS group on day 180. The tissue suspension was centrifuged for 10 min at 600 × *g*, and the supernatant was discarded. After washing with the collection medium three times (Kaneko et al., 2013; Kaneko, Kikuchi, Men, et al., 2017; Kaneko, Kikuchi, Nakai, et al., 2017), the pellet was resuspended in a small volume of collection medium and maintained at room temperature until used for ICSI. Sperm obtained from each host mouse were used separately for ICSI.

### 2.6 Oocyte maturation

Porcine cumulus‐oocyte complexes (COCs) collected from ovaries at a local abattoir were matured in vitro in North Carolina State University (NCSU)‐37 solution (Petters & Wells, 1993) with modifications (Kikuchi et al., 2002). After in vitro maturation culture for 44–46 hr, expanded COCs were denuded of their cumulus cells mechanically by gentle pipetting after brief treatment with 150 IU/ml hyaluronidase: oocytes showing extrusion of the first polar body were harvested as mature oocytes.

### 2.7 ICSI and oocyte stimulation

ICSI was performed in accordance with our previous studies (Kaneko et al., 2013; Kaneko, Kikuchi, Men, et al., 2017; Kaneko, Kikuchi, Nakai, et al., 2017; Men et al., 2016; Nakai et al., 2003, 2006, 2007, 2009, 2010). Approximately 20 in vitro‐matured oocytes were transferred to a 20‐μl drop of IVC‐PyrLac supplemented with 20 mmol/L HEPES (IVCPyrLac ‐HEPES) (Kaneko et al., 2013; Kaneko, Kikuchi, Men, et al., 2017; Kaneko, Kikuchi, Nakai, et al., 2017; Men et al., 2016; Nakai et al., 2003, 2006, 2007, 2009, 2010) that had been placed on the cover of a plastic dish (Falcon 351005, Thermo Fisher Scientific). A small volume (0.5 μl) of the sperm suspension was transferred to a 2‐μl drop of IVC‐PyrLac‐HEPES supplemented with 4% (w/v) PVP (Mr 360,000) that had been placed close to the drops containing the oocytes. These drops were covered with paraffin oil (Paraffin Liquid; Nakarai Tesque, Kyoto, Japan). A morphologically normal single sperm was aspirated tail first from the suspension and injected into the ooplasm of the mature oocyte using a piezo‐actuated micromanipulator (Prime Tech, Tsuchiura, Japan) (Kaneko et al., 2013; Kaneko, Kikuchi, Men, et al., 2017; Kaneko, Kikuchi, Nakai, et al., 2017; Men et al., 2016; Nakai et al., 2009, 2010). One hour after the injection, the sperm‐injected oocytes (20 in all) were transferred to an activation solution consisting of 0.28 mol/L D‐mannitol, 0.05 mmol/L CaCl_2_, 0.1 mmol/L MgSO_4_, and 0.1 mg/ml BSA. They were then stimulated with a direct current pulse of 1.5 kV/cm for 60 μs using an electric fusion generator (ECFG21, Nepagene, Ichikawa, Japan). As a negative control, parthenogenetic oocytes were used: 15 oocytes were stimulated with an electrical pulse under the same conditions (1.5 kV/cm for 60 μs) as those for the ICSI groups without pretreatment with an injection pipette (parthenogenetic group). The above procedures were carried out separately on sperm obtained from two host mice (2 replications).

### 2.8 Assessment of the developmental ability of sperm‐injected oocytes

In this study, the developmental competence of oocytes that had been injected with xenogeneic sperm was assessed by in vitro culture to the blastocyst stage. Sperm‐injected oocytes or parthenogenetic oocytes were cultured for 6 days, as described previously (Kikuchi et al., 2002). They were then fixed with acetic alcohol (acetic acid‐to‐ethanol = 1:3, v/v) and stained with 1% aceto‐orcein. Any embryo with an apparent blastocele consisting of more than 10 cells was defined as a blastocyst. The rate of blastocyst formation and the total number of cells in the blastocysts were assessed.

### 2.9 Histological analyses

Testicular fragments before transplantation and those excised from the grafts in mice were embedded in paraffin, and then cut into sections 6 μm thick and stained with hematoxylin and eosin. The seminiferous cord/tubule cross‐sections were then sorted into the followingcategories, as described previously (Kaneko et al., 2008, 2012, 2013, 2014; Kaneko, Kikuchi, Men, et al., 2017; Kaneko, Kikuchi, Nakai, et al., 2017): (a) no germ cells present (cord/tubule cross‐sections showing Sertoli cells only); (b) gonocytes or spermatogonia present; (c) spermatocytes present as the most advanced germ cells; (d) round spermatids present as the most advanced germ cells; (e) elongated spermatids present as the most advanced germ cells; and (f) mature sperm (spermatozoa) present in the lumina of the tubules in which elongated spermatids coexisted. All seminiferous cord/tubule cross‐sections observed in one section of each testicular tissue were categorized. For analysis of the testicular fragments at day 0 (just before grafting), the data obtained using 5–8 fragments from each piglet were pooled and the percentages calculated. Mean (±*SEM)* percentages were calculated using the data obtained from 5 MS and 4 WC piglets respectively: the values were expressed as those per MS or WC piglet. For assessment of the differentiation of the recovered grafts between days 60–180, the data obtained using 3 fragments from each mouse were pooled and the percentages calculated. Mean (±*SEM*) percentages were calculated using the data obtained from 4 host mice: the values were expressed as those per mouse.

### 2.10 Statistical analyses

The mean weight of four sets of 25 fragments before grafting was 23.6 ± 2.0 (±*SEM*) mg for MS piglets and 25.8 ± 0.5 for WC piglets. When the total weight of grafted tissues recovered from a single mouse exceeded the 95% confidence limit (19.6–27.6 mg for MS grafts and 24.8–26.8 mg for WC grafts), porcine testicular tissues were judged to have grown in the mouse, and data obtained from such mice were subjected to statistical analyses. The effects of donor strains and time after grafting on testis development were compared between the groups using two‐way ANOVA. Testis development in relation to time after grafting in each group was examined using one‐way ANOVA. When a significant effect was detected by ANOVA, the difference between two means was determined by Student's *t* test and differences among more than two means were determined by Tukey's test. The General Linear Models of Statistical Analysis Systems, ver 9.4 (SAS Inc., Cary, NC, USA), was used for these analyses. Data are expressed as mean* *± *SEM* unless otherwise indicated. Differences at *p *< 0.05 were considered significant.

## 3 RESULTS

### 3.1 Growth of testicular tissue

**Figure 1:**
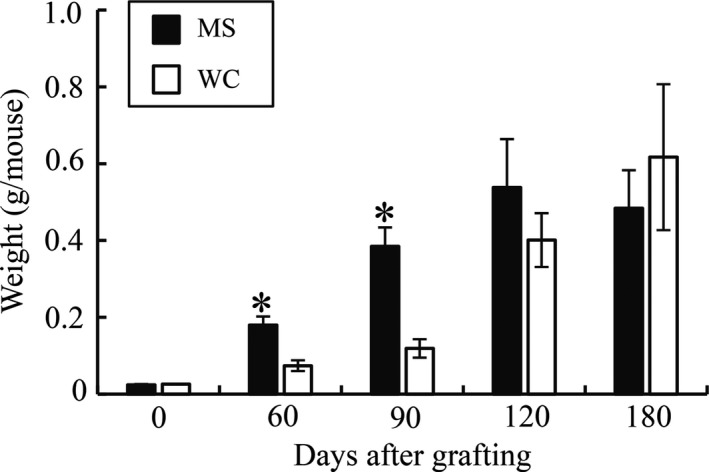
Weights of cryopreserved testicular tissue from Meishan (MS) and Western crossbreed (WC) piglets in host mice. All visible grafts were recovered from the mice, and the values are means (±*SEM*) of the total weight per mouse for 6–8 mice in each group. Asterisks indicate significant (*p *< 0.05) differences in the weights of testicular grafts between the MS and WS groups

Growth of porcine testicular tissue, judged using the criteria explained in the data analysis section was recorded in all of the host mice (*n* = 28) in the MS group and in 24 out of 26 mice in the WC group (Table 1). The weights of all visible grafted tissues per mouse increased (*p *< 0.05) with time after grafting in each group. However, those in the MS group were higher (*p *< 0.05) than in the WC group between days 60 and 90 (Figure 1). After day 120, there were no significant differences in graft weights between the two groups (*p *> 0.1).

**Table 1:** Number of host mice in which porcine testicular grafts gained weight and released sperm into the collection medium after being minced into small pieces

	No. of mice^b^ in each group^c^	
Day of recovery^a^	MS group	WC group
60	7 (2)	6 (0)
90	8 (8)	6 (0)
120	7 (7)	6 (1)
180	6 (6)	6 (6)

a Porcine testicular xenografts were recovered from host mice from day 60 to 180 (day 0 = grafting).

b Number of host mice harboring grown xenografts followed by the number of host mice in which porcine testicular grafts released sperm (in parenthesis).

c Mice in the MS and WC groups received testicular tissues from Meishan and Western crossbred piglets, respectively.

### 3.2 Differentiation of seminiferous tubules

**Figure 2:**
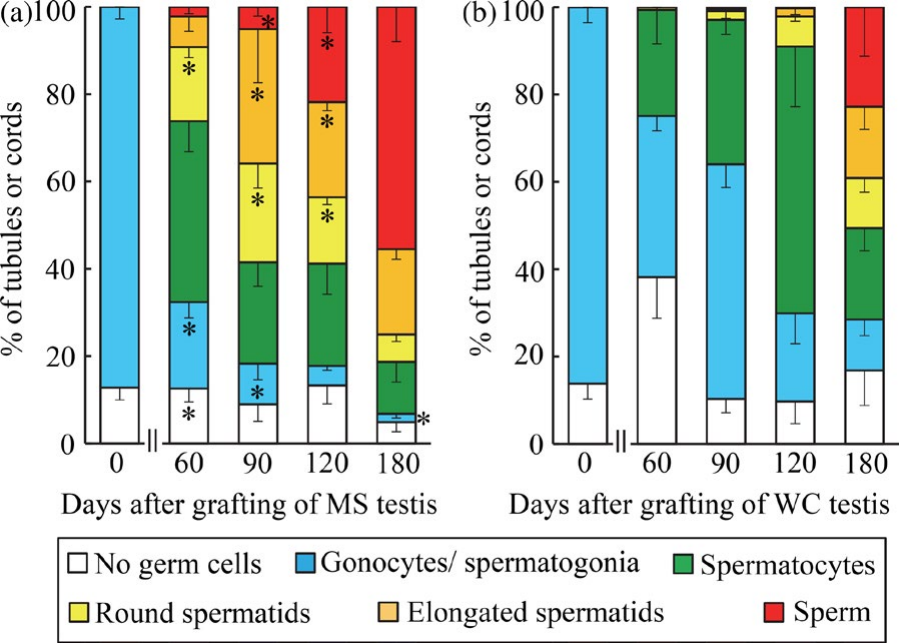
Differentiation of seminiferous cords/tubules in testicular tissue of (a) Meishan (MS) and (b) Western crossbreed (WC) pigs recovered from host mice, as classified by the most advanced type of germ cell present. Values on day 0 are mean ± *SEM* per piglet (*n* = 5 MS and *n* = 4 WC piglets). Values between days 60 and 180 are mean* *± *SEM* per mouse (*n* = 4 mice). Asterisks indicate significant differences (*p *< 0.05) in the percentages of each categorized cord/tubule between the MS and WS groups

**Figure 3:**
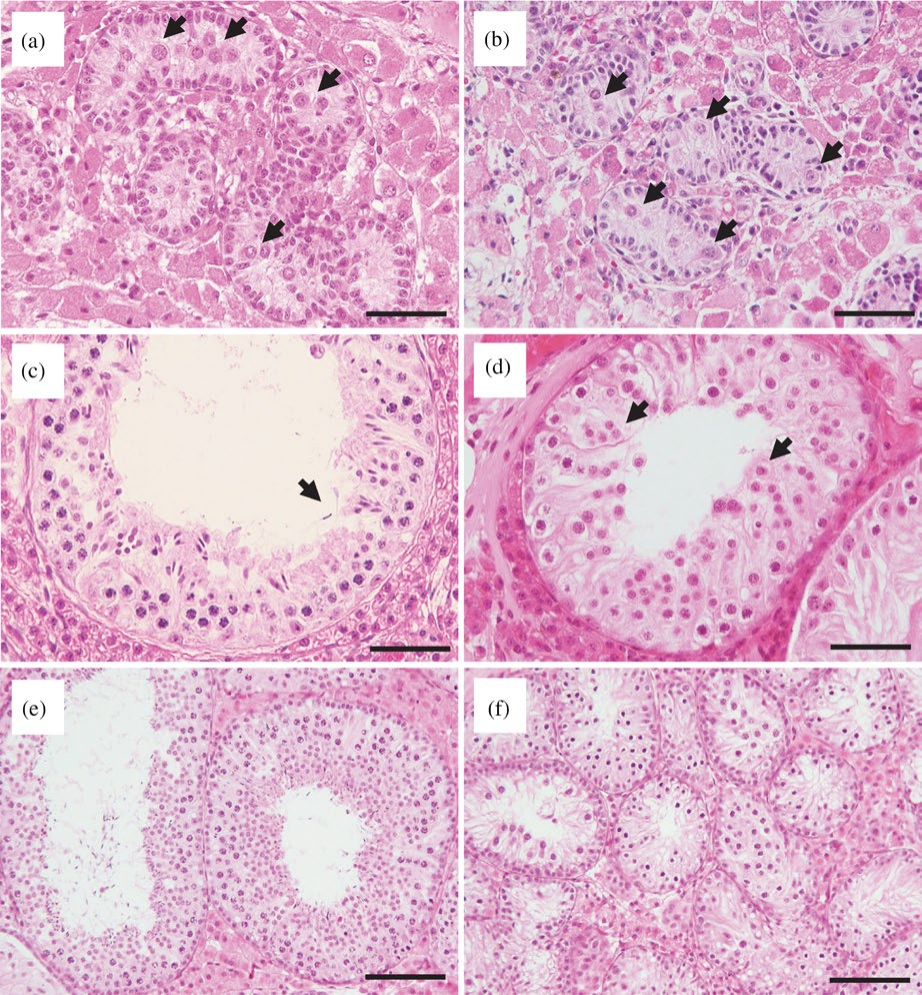
Histological appearance of cryopreserved testicular tissue from Meishan (MS) and Western crossbreed (WC) piglets recovered from host mice. Cryopreserved fragments obtained from testes of neonatal (a) MS and (b) WC pigs after warming (just before grafting). Grafts of (c) MS and (d) WC pigs obtained from mice on day 60 and grafts of (e) MS and (f) WS pigs on day 120. Arrowheads indicate gonocytes in (a) and (b), sperm in (c) and round spermatids in (d). Scale bars represent 50 μm in the images from (a) to (d) and100 μm in (e) and (f)

In the testicular tissue before grafting (Day 0), more than 85% of seminiferous cords contained only gonocytes/spermatogonia and Sertoli cells in the MS and WC piglets (Figures 2, 3a and 3b). Sixty days after grafting, testicular grafts in the MS group had already developed elongated spermatids or sperm (Figure 3c): the proportions of seminiferous tubule cross‐sections containing these cell types were 7.0 ± 3.4% and 2.1 ± 1.6%, respectively (Figure 2a). At the same sampling point, grafts in the WC group showed a very low proportion (0.6 ± 0.5%) of tubules containing round spermatids as the most advanced cell type (Figures 2b and 3d). From days 90 to 120, the proportion of tubule cross‐sections containing round spermatids, elongated spermatids or sperm was higher (*p *< 0.05) in the MS group than in the WC group, whose sperm were first detected on day 120 (Figure 2b). In the MS grafts on day 120, tubule cross‐sections containing elongated spermatids or sperm occupied 43% of the total tubules (Figures 2a and 3e), while in the WC grafts, the ratio was only 2%, although tubules containing spermatocytes were predominant (61.1 ± 13.8%, Figures 2b and 3f). On day 180, there were no significant differences (*p *> 0.08) between the two breeds in the proportion of tubules in each category, except for that of gonocytes/spermatogonia (Figure 2).

### 3.3 Recovery of sperm

**Figure 4:**
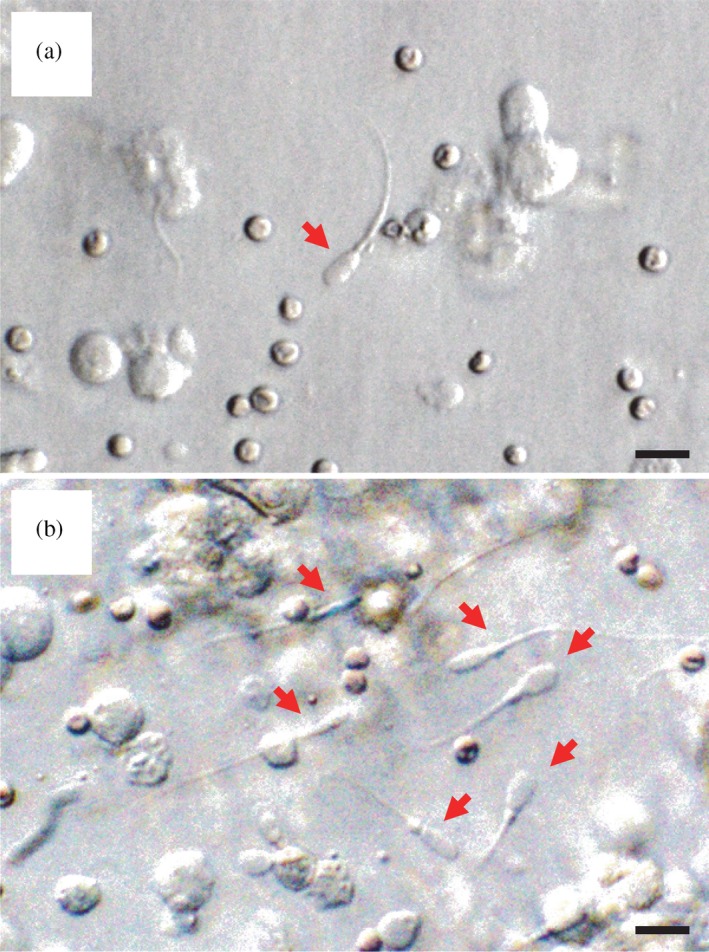
Representative images of sperm (arrows) retrieved from Meishan xenografts (MS‐group) on days (a) 60 and (b) 180. Scale bars represent 10 μm

Sperm were recovered from grafts in 2 out of 7 mice in the MS group on day 60 (Table 1 and Figure 4a): the recovery rate reached 100% on day 90 and later (Figure 4b). On the other hand, in the WC group, sperm were recovered from 1 of 6 mice on day 120 and the ratio increased to 100% on day 180 (Table 1). Most of the sperm in the MS group were morphologically normal and often had cytoplasmic droplets (Figure 4). Sperm in the WC group were morphologically similar to those obtained from the MS grafts.

### 3.4 Developmental ability of Meishan xenogeneic sperm

**Figure 5:**
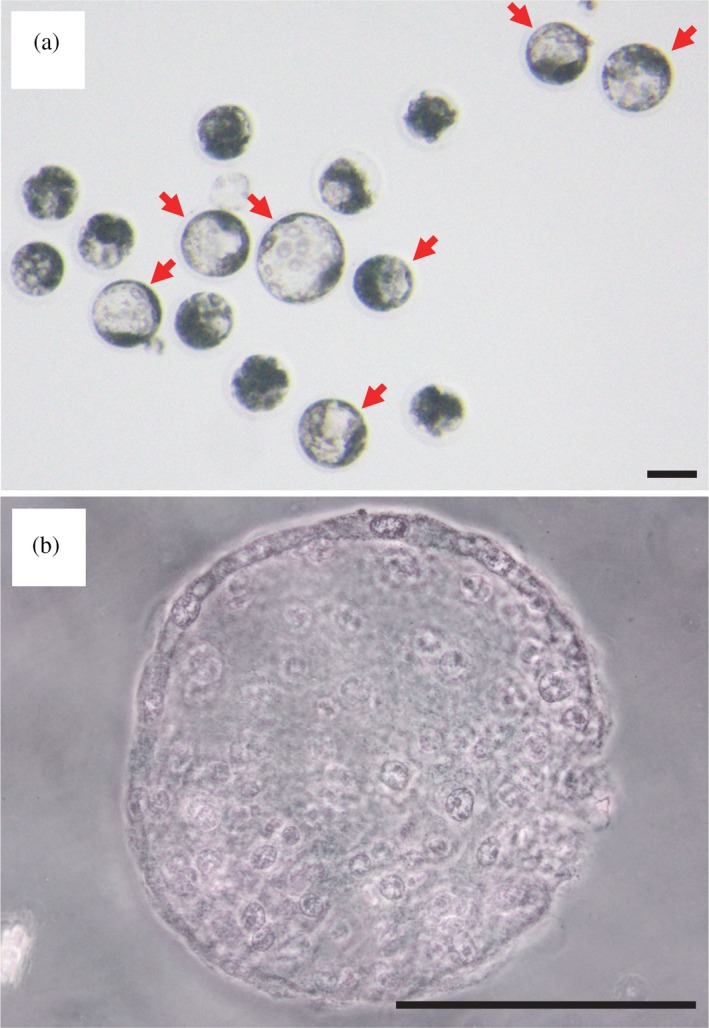
Blastocyst formation from sperm‐injected oocytes. (a) An example of porcine blastocysts obtained by ICSI using sperm obtained from Meishan testicular xenografts. Arrows indicate blastocysts. (b) An example of a well‐developed blastocyst with 145 cells. Scale bars represent 100 μm

The in vitro developmental ability of oocytes that had been injected with sperm obtained from Meishan testicular xenografts, or activated parthenogenetically, is shown in Table 2. Percentage blastocyst formation by ICSI using Meishan xenogeneic sperm (ICSI group) was approximately 15% (Figure 5a), whereas that after parthenogenetic activation was 7% (parthenogenetic group). The total number of cells per blastocyst was 61.4 ± 7.4 in the ICSI group (Table 2 and Figure 5b), which was not significantly different from that in the parthenogenetic group.

**Table 2:** In vitro development of porcine oocytes injected with sperm obtained from Meishan testicular xenografts

Experimental group	No. of mature oocytes used	No. (%) of blastocysts formed	No. of cells per blastocyst (range)
ICSI group^a^	123	18 (14.6)	61.4 ± 7.4 (24–145)^c^
Parthenogenetic group^b^	30	2 (6.7)	46.5 ± 18.5 (28, 65)^d^

a Electrical stimulation after sperm injection.

b Electrical stimulation without the injection procedures.

c Value is the mean ± *SEM* followed by the range of the number of cells in individual blastocysts (parenthesis).

d Value is the mean ± *SEM* followed by the number of cells in two blastocysts (parenthesis).

## 4 DISCUSSION

Indigenous pigs are expected to be a reservoir of unique genetic diversity (Ishihara et al., 2018), since they have adapted to geographically isolated environments for many years. According to a report from the Food and Agriculture Organization of the United Nations (FAO), 14% of pig breeds are categorized as being at risk for continuance (Baumung & Wieczorek, 2015). Preservation and utilization of germplasm from indigenous pigs is valuable for conserving the genetic diversity of pig species. In order to utilize neonatal testis for the conservation of indigenous pigs, it is necessary to know whether their testicular tissue, after cryopreservation, can acquire the capacity to produce sperm by xenografting, and whether these xenogeneic sperm have developmental competence. The findings of the present study clearly indicate that cryopreserved testicular tissue obtained from indigenous Meishan piglets can produce sperm in host mice, and that these xenogeneic sperm have the ability to generate blastocysts.

In the present study, MS grafts gained weight faster than WC grafts between days 60 and 90, but this tendency disappeared after day 120. These findings are consistent with previous studies that have assessed in situ testicular growth in both breeds: Testicular weight increased more rapidly in Meishan than in Western crossbreed boars between 70 and 112 days of age (Ford & Wise, 2009), but at around 6 months of age, Meishan testis had a lower weight (252 g/paired testis weight, Harayama et al., 1991) than Western breed testis (320–564 g/paired testis weight, Allrich, Christenson, Ford, & Zimmerman, 1983; FlorCruz & Lapwood, 1978). In addition, histological examination revealed that differentiation of seminiferous cord/tubules in MS grafts was more promoted than that in WC grafts until 120 days after xenografting. Onset of sperm production defined as the appearance of sperm in the seminiferous tubules was evident at day 60 in the MS group, but at day 120 in the WC group, as reported in our previous study (Kaneko et al., 2013). The percentages of tubule cross‐sections containing elongated spermatids or sperm were higher in the MS group than in the WC group from days 90–120 (36–43% in the MS group and 0.8–2% in the WS group). Thus, even in the same milieu (i.e., nude mice), Meishan testicular xenografts showed more rapid growth and differentiation than Western breed grafts, a characteristic that appears to be inherent in Meishan testis.

Our vitrification protocol (Dinnyés et al., 2000; Kaneko et al., 2013; Kaneko, Kikuchi, Men, et al., 2017; Kaneko, Kikuchi, Nakai, et al., 2017; Somfai et al., 2007) using EG, PVP and trehalose as cryoprotectants has proven to be useful for ultra‐rapid cooling of Meishan testicular tissues, as well as testes of Western breeds (Kaneko et al., 2013; Kaneko, Kikuchi, Men, et al., 2017; Kaneko, Kikuchi, Nakai, et al., 2017), oocytes (Somfai et al., 2010) and zygotes (Somfai et al., 2009). Moreover, the present WC grafts, that had been vitrified and stored in liquid nitrogen for more than 5 years, retained the ability to produce sperm, similar to grafts after short‐term storage (Kaneko et al., 2013). This suggests that storage of gonadal tissue for periods of up to 10 years after vitrification would be possible.

Development of fertilized oocytes to the blastocyst stage is an important result indicating that further development to piglets might be possible. We therefore examined the developmental ability of Meishan xenogeneic sperm by injecting them into mature oocytes, and these sperm‐injected oocytes developed to the blastocyst stage after 6 days of culture. The quality of the present blastocysts (total number of cells) was similar to that in previous studies (Kikuchi et al., 2002; Somfai et al., 2009) where live piglets were obtained after in vitro fertilization. Our previous ICSI using xenogeneic sperm obtained from Western crossbreed piglets generated live piglets (Kaneko et al., 2013; Kaneko, Kikuchi, Nakai, et al., 2017; Nakai et al., 2010). Moreover, the rate of blastocyst formation was 14.6% in the ICSI group, compared with 6.7% in the parthenogenetic group. Considering the above findings, we can infer that at least half of the blastocysts obtained by ICSI resulted from fertilization with sperm obtained from xenografted Meishan tissue. Thus, xenogeneic Meishan sperm are likely to have the ability to support embryonic development to the blastocyst stage.

In summary, we have cryopreserved testicular tissues obtained from neonatal Meishan boars and transplanted them into nude mice. The Meishan xenografts grew and differentiated faster than xenografts from Western crossbreed piglets and produced morphologically normal sperm. Sperm retrieved from porcine xenografts were proven to have the ability to generate blastocysts after ICSI. Testicular tissue from indigenous pigs can provide a genetic reservoir enabling conservation of rare breeds.
